# Preferred place of death for children and young people with life-limiting and life-threatening conditions: A systematic review of the literature and recommendations for future inquiry and policy

**DOI:** 10.1177/0269216313483186

**Published:** 2013-09

**Authors:** Myra Bluebond-Langner, Emma Beecham, Bridget Candy, Richard Langner, Louise Jones

**Affiliations:** Louis Dundas Centre for Children’s Palliative Care, UCL Institute of Child Health, London, UK; Department of Sociology, Anthropology and Criminal Justice, Rutgers University, Camden, NJ, USA; Marie Curie Palliative Care Research Unit, UCL Mental Health Sciences Unit, University College Medical School, London, UK; Louis Dundas Centre for Children’s Palliative Care, UCL Institute of Child Health, London, UK; Marie Curie Palliative Care Research Unit, UCL Mental Health Sciences Unit, University College Medical School, London, UK; Louis Dundas Centre for Children’s Palliative Care, UCL Institute of Child Health, London, UK; Marie Curie Palliative Care Research Unit, UCL Mental Health Sciences Unit, University College Medical School, London, UK

**Keywords:** Paediatrics, palliative care, terminal care, preference, location of death, systematic review

## Abstract

**Background:**

Home is often cited as preferred place of death in the United Kingdom and elsewhere. This position, however, usually relies on data concerning adults and not evidence about children. The latter data are scant, primarily retrospective and from parents.

**Aim:**

To review the literature on preference for place of death for children and young people with life-limiting or life-threatening illnesses.

**Design and data sources:**

The databases MEDLINE, CINAHL and EMBASE were searched from 2004 to 2012, as well as bibliography, key author and grey literature searches. Policy documents, empirical, theoretical and peer-reviewed studies and conference abstracts were included. Articles were assessed for study quality.

**Results:**

Nine studies were included from five countries. Six reported a majority of parents (only one study interviewed adolescents) expressing preference for death at home. Other studies differed significantly in their findings; one reporting 35.1% and another 0% preferring death at home. Some parents did not express a preference. Six of the studies included only parents of children who died from cancer while being treated at tertiary centres that offered palliative care services. Such results cannot be generalised to the population of all life-limiting and life-threatening illnesses. Furthermore, the methods of the studies reviewed failed to accommodate the full range and dynamic character of preference.

**Conclusion:**

The evidence base for current policies that stress the need to increase home death rates for children and young people with life-limiting and life-threatening conditions is inadequate. Further rigorous research should collect data from parents, children and siblings.

## Background

In the United Kingdom and mainland Europe, there is an increasing emphasis on the desirability of facilitation of choice for patients and carers in healthcare, including a focus on facilitating care at home.^1^ For those approaching the end of their life, the opportunity for care at home and a home death is strongly advocated by policymakers.^2^ In the United Kingdom, especially, there is a recent drive to maximise the amount of care in the home.^3,4^ This is the case for both adults and for children and young people (CYP). Documents include statements such as ‘Children often want to be at home and families usually want to keep them at home through illness and death’ (p. 3).^2^ Similarly, the *Independent Review of Palliative Care* states, ‘Most families would like their child to be supported in dying at home’ (p. 4).^4^

With respect to children, these beliefs, and in turn, these policies, are not supported by robust evidence gathered from CYP, their families or healthcare professionals (HCP). Knowledge has been extrapolated from recommendations for adults to the care of CYP without understanding differences in care and preferences between adults and children and their families.^5,6^ The limited research that has been conducted is highlighted as problematic by previous articles.^7–10^ Most studies are retrospective and have involved interviews with bereaved parents or HCP. In this article, we report a comprehensive modified systematic review of the literature on preference on place of death (POD) for CYP with life-limiting conditions (LLCs) and life-threatening illnesses (LTIs).

## Methods

### Inclusion/exclusion criteria

Studies that reported quantitative data on preference for POD or report data that allowed the calculation of a numeric finding or make a statement that could be paraphrased in numeric terms such as ‘*most* parents preferred’ were included. English language policy documents, empirical, theoretical and peer-reviewed studies and conference abstracts published between 2004 and 2012 were included; book chapters and personal opinion pieces were excluded. Studies published before 2004 were not considered, as a comprehensive review of research evidence in palliative care was conducted in 2004 to accompany the UK National Institute for Health and Clinical Excellence (NICE) guidance on supportive and palliative care for adults with cancer.^11^ This date also corresponds with the development of the Association of Children’s Palliative Care (ACT) pathway,^12^ which sets a framework for providers and commissioners of care for CYP.

### Data sources

The databases MEDLINE, CINAHL and EMBASE were searched in May 2012. In addition to running the search strings in each database, we conducted hand searches, including forward and backward citation searches of shortlisted studies, recommendations from experts and finally, the grey literature (including reports and policy documents). Key websites such as the Department of Health and the Together for Short Lives websites were searched to retrieve published work missed through the other searches.

Two review authors (E.B. and L.J.) screened citations against the selection criteria. Following screening, the same authors assessed the full text of potentially eligible citations for inclusion. Key information was extracted from the studies including population, disease and the methodology of the study. We aimed, if homogeneity and quality were satisfactory, to combine the data in the studies to provide an overall measure of preference.

For the qualitative study, quality was assessed using recommendations outlined by the Critical Appraisal Skills Programme tools.^13^ For quantitative studies, a scale developed from Guyatt et al.^14^ was used, and for the mixed-methods study, a scale developed by Pluye et al.^15^ was used.

### Search strategy

Search terms were used for each of the following components:

1. Death including terminal* or palliative or dying or death or die or deteriorat*.
2. Child including infant or infan* or newborn or newborn* or new-born* (a predefined search string used in a Cochrane review provided an already validated comprehensive search string for this component of the search).
3. Place including home* or hospital* or hospice*.
4. Prefer*.

Full details are available from the authors.

## Results

**Figure 1.**
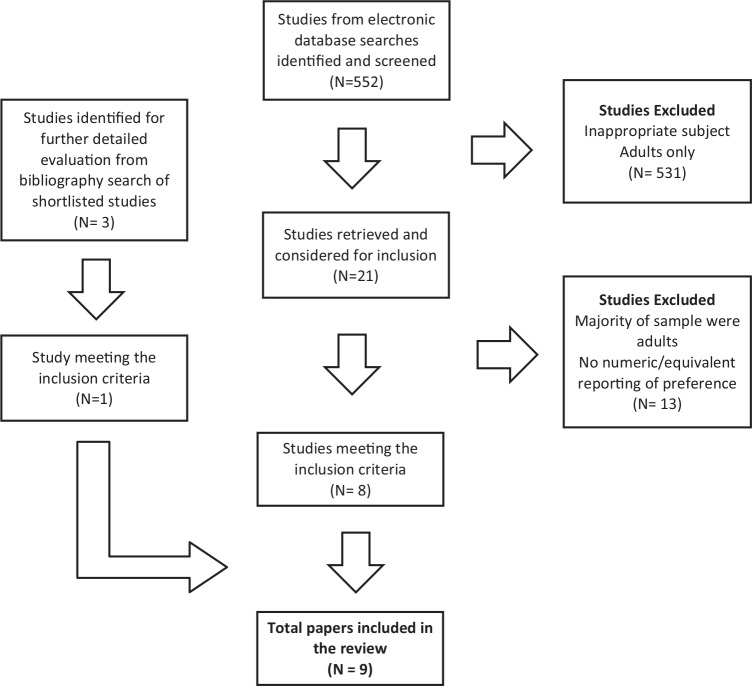
Flow of articles through review.

The search strategy generated 552 studies, excluding duplicates. E.B. and L.J. screened the 552 abstracts and retained 21 studies that required the full article to screen. Studies were excluded if they were on an inappropriate subject (not on POD in CYP entirely) or clearly only about adults. Three articles were found from hand searching. Of these, only one article met the inclusion criteria.^16^ In total, nine articles met our inclusion criteria and were included in the review^16–24^ (see Figure 1). For description of included articles, see Table 1.

**Table 1.** Summary of articles in systematic literature review.

Source	Population (country; *N*)	Age (years)	Disease	Methodology	Quality assessment score
Dussel et al.^20^	USA; *N* = 140	‘Children’	Cancer	Retrospective chart review, interviews and surveys	Medium
				Parent report	
Grinyer and Thomas^24^	UK based (but one parent from Australia and one from Germany took part); *N* = 13	18–25	Cancer	Retrospective narrative correspondence approach	Medium
				Parent report	
Heath et al.^17^	Australia; *N* = 96	‘Children’ (mean age at death 9.4 years)	Cancer	Retrospective structured interview and self-report questionnaire	Medium
				Parent report	
Hechler et al.^16^	Germany; *N* = 56 parents (of 48 children)	0–20	Cancer	Retrospective structured interview	Medium
				Parent report	
Lyon et al.^18^	USA; *N* = 80 (40 YP/family surrogate dyads)	14–21	HIV	Prospective survey YP/parent report	N/A (abstract only)
Montel et al.^21^	France; *N* = 38 parents (from 21 families)	15–25	Cancer	Retrospective mixed-methods, interviews and chart review	Medium
				Parent report	
Siden et al.^22^	International (Australia. Canada, United Kingdom); *N* = 703	‘Children’	Range of life-limiting/threatening illnesses	Retrospective chart/database review N/A (chart review)	Medium
Vickers et al.^23^	United Kingdom; *N* = 164 (155 with complete records)	0–19	Cancer	Prospective surveys ‘Family’ report	Medium
Wolff et al.^19^	Germany; *N* = 51	‘Children’	Range of life-limiting/threatening illnesses	Retrospective survey	Medium
				Parent report	

YP: young people.

### Methods

All nine articles reported primary research. Of these, seven were quantitative,^16–20,22,23^ one was mixed methods^21^ and one was qualitative.^24^ Seven collected data retrospectively^16,17,19–22,24^ and two prospectively.^18,23^ The quantitative studies were chart reviews, analyses of medical records and death certificates, or surveys. The qualitative study used a narrative correspondence approach. The mixed-methods study combined semi-structured interviews with a chart review. Six articles reported data collected from parents,^16,17,19–21,24^ two from a mix of parents and young people (YP)^18,23^ and one was a chart review, data taken from information the clinician had previously recorded.^22^

### Populations in articles included

Four of the studies reported their sample to be ‘children’ (all parent proxy apart from the one chart review, which was information recorded by the clinician), three studies were in the ‘YP’ age bracket, 14–21, 15–25 and 18–25 years old, respectively (two parent proxies and one YP and parent reporting) and two studies had a wide range of CYP of 0–19 and 0–20 years old (one ‘family’ reporting and one parent proxy).

Studies overall reported findings for slightly more male CYP than female CYP. Studies were conducted in the United Sates (2 studies),^18,20^ United Kingdom (2 studies),^23,24^ Germany (2 studies),^16,19^ France (1 study),^21^ International (Australia, United Kingdom and Canada) (1 study)^22^ and Australia (1 study).^17^

### Disease

Six articles focused on cancer,^16,17,20,21,23,24^ two articles focused on a heterogeneous range of LTIs and LLCs^19,22^ and one article focused on HIV.^18^

### Methodological quality of included studies

#### Quality assessment (see Table 1)

Study quality was assessed for eight of the nine studies^16,17,19–24^ by two researchers (E.B. and P.K.), and any disagreements were resolved by consensus. One research paper was a conference abstract^18^ and therefore lacked sufficient detail to assess quality. All eight studies assessed were rated as medium-quality studies (out of a possible ranking of high, medium or poor quality). The studies lacked quality due to the lack of explanation of the methods or analysis sections or because of small sample sizes.

### Findings

#### Preference for home

Nine studies report on preferences for death at home or report data from which we inferred preference. Six of these studies focused on children with cancer.^16,17,20,21,23,24^ Five of the six were retrospective studies interviewing parents of children who had died.^16,17,20,21,24^ Vickers et al.^23^ found that at entry to the study, that is, when it was thought that cure was no longer possible, 98 (68%) of 164 families recorded a preference for home as POD. In the last month of life (or at entry to the study for the 28 children who died during the first month), the preference for home death rose to 132 (80%) of 164. Hechler et al.^16^ report that 88% (*N* = 48) of the families interviewed chose, in hindsight, home as the most ‘appropriate’ POD. In Grinyer and Thomas’^24^ narrative correspondence study of the preferences of adolescents as reported by their parents, it was found that the ‘majority’ of the 13 subjects preferred a death at home, ‘two-thirds of the 13 young adults … were able to die at home, and one wished to do so but died in a hospice’ (p. 127). We have inferred from Dussel et al.’s^20^ findings that since at least 67 (48%) of 140 families whose child died at home would not, in retrospect, have preferred a death in another location at least 48% would have preferred a home death. Heath et al.^17^ reported that of the families who said that they had time to plan where their child would die (*N* = 61, 63%), 89% (*N* = 54) said they preferred to have their child die at home (56% of the total *N*).

Lyon et al.^18^ surveyed US adolescents with HIV. They found that 60% (*N* = 40) of subjects chose home as preferred POD. Two studies dealt with populations with a range of illnesses including both cancer and progressive LLC and LTI.^19,22^ Wolff et al.^19^ report that 69% (*N* = 35) of families preferred that their child be at home at end of life. This figure was inferred by Wolff et al.^19^ from the actual POD: ‘most families preferred their child to be at home as shown by the reduced frequency of children dying in the hospital’ (p. 281). Siden et al.^22^ also relied upon actual location of death as reflecting a choice on the part of families. They report an overall choice of home at 35.1% (*N* = 703). Across the three sites studied, the findings for home death were Westmead (Australia) = 42.4%, Canuck Place (Canada) = 25% and Martin House (United Kingdom) = 34.6%.

#### Preference for hospital

Not all the nine studies reported a finding on preference for locations other than home or reported findings that would have allowed preferences other than home to be inferred. Vickers et al.^23^ reported that for those children who survived beyond the first month of the study, no families planned a hospital death. Families of the 4 of the 38 children who died within 30 days of entry planned a hospital death. Montel et al.^21^ found that in retrospect, all the families preferred hospital as the POD. Grinyer and Thomas^24^ report that two young adults in this study died in hospital but preference is not reported. From Dussel et al.’s^20^ findings, we inferred that 47 of 140 (34%) families who experienced a hospital death would not in retrospect prefer a different location.

Lyon et al.^18^ found that 15% of the adolescents in this study expressed a preference for hospital as POD. Siden et al.^22^ report an average of 31.9% of the cases studied showing a preference for hospital death across three study sites. Locally, the preferences were 39.1% in Australia, 13.8% in Canada and 35.2% in the United Kingdom.

#### Preference for hospice

There are no findings in three of the studies^16,19,21^ about preference for hospice, and no inferences can be drawn from Dussel et al.’s^20^ data. Vickers et al.^23^ found that no parents planned or preferred a hospice death at entry to the study; however, four indicated a preference for death in a children’s hospice in the last month of life. The authors report that in the study population, ‘only a small number’ (p. 4475)^23^ of children were reported to have spent time in a hospice at any point in their illness.

Siden et al.^22^ found the average for preference for hospice across the three sites to be 32.1%. By location, the breakdown was Australia = 18.5%, Canada = 58.6% and United Kingdom = 29.6%.

Lyon et al.^18^ found that 5% of the adolescents surveyed expressed a preference for death in a hospice; 68% of the adolescents had never heard of hospice. Of those who had heard of hospice, 25% wanted hospice involvement if they were dying.

In Grinyer and Thomas’ study,^24^ two deaths occurred in hospice. In one case, it was reported by the parent that the adolescent’s preference was to be at home but this was thought to be not possible because of the YP’s condition.

#### No preference

Two studies^18,23^ report on subjects who express no preference. Vickers et al.^23^ reported that 24 families out of 126 were undecided about planned POD at entry to the study and 17 remained so at the last month of life. In Lyon et al.’s^18^ study of adolescents, 20% of those surveyed gave no preference for POD.

#### Change of preference over time

Vickers et al.^23^ provide data for preference at both entry to study and at the last month of life. The number of those preferring home death increased from 98 to 120, the number preferring hospice increased from 0 to 4 and the number preferring hospital death remained constant.

Siden et al.^22^ make the relevant comment:

Families had the opportunity to move back and forth between settings, for example, they may have chosen home for end-of-life care initially but later opted to move to a hospice. In our clinical experience, some families made more than one such move as their situation changed. (p. 832)

This is, however, a clinical observation rather than part of the study data.

## Discussion

This article set out to review the literature on preference for POD in CYP with LLC and LTI. Nine studies on preference for POD were identified. The studies reviewed have limitations of both population selection and method, which preclude synthesising their findings or generalising from any one of them to the subject population, LLC and LTI, as a whole. There is, then, no clear evidence about the preference for POD for children with LLC and LTI.

### Population bias

Six of the nine studies focus exclusively on children and young adults who died of cancer while cared for by a specialist oncology team and five of the six at a tertiary paediatric facility. In the two studies of populations with a broader range of progressive LLC and LTI (Siden et al.^22^ and Wolff et al.^19^), 37% and 57%, respectively, had cancer. Compare this with Fraser et al.’s^25^ recent findings that for LLC (conditions for which there is no cure and which will become fatal) in England 13.7% are oncologic. Siden et al.^22^ found that children with cancer were significantly more likely than other disease populations to die at home (with the exception of metabolic diseases). Indeed, this relation is well documented.^26^ The obvious question is whether the care required by most non-haematological cancers is more compatible with care and death at home. Oncology is also a place where home outreach is extensively developed.

In six of the studies,^16,17,19,21–23^ palliative care teams were available to families, and more or less extensive home care was available as well. In three of these,^19,22,23^ the sample was simply the population treated by the palliative care team.

Populations that consist wholly or largely of families referred to specialist palliative care are somewhat atypical within the larger population of children with LTIs and LLCs. In spite of the many statements by professional bodies that urge early and widespread involvement with palliative care, this is not the reality. Referrals typically are too few and late in the disease trajectory. Physicians tend to refer families to palliative care only if they think the family is willing or able to deal somewhat openly with their child’s impending death. Thus, the factors that might predispose a family to home death lead them into the study population itself.

Lowton^27^ points out that with non-cancer diseases such as cystic fibrosis (CF), opposing pathways are routinely pursued simultaneously: ‘[P]reventative, restorative and palliative treatments [are] administered in tandem throughout the lifecourse’ (p. 1057). These ideas may have long been understood by families receiving care for their children with LLC and LTI for whom optimum care of all varieties is actively sought – ongoing attempts at prolonging life alongside maximum symptom control. The uncertain, unpredictable disease course makes engagement with palliative care difficult. Hence, we should not infer that studies of preference that focus on a single disease population or in which a disease like cancer is overrepresented can be generalised to apply to all LLC and LTI.

### Demographics

Are the individuals surveyed representative of the larger populations within which they are situated with regard to, for example, sex, race, education? Both Dussel et al.^20^ and Heath et al.^17^ note the homogeneity of their samples – mainly White, middle class^20^ and only English speaking.^17^ In the two studies for which data on gender is clear, mothers were the overwhelming parent interviewed: 82% in Heath et al.^17^ and at least 77% in Hechler et al.^16^ This is consistent with the findings of Macdonald et al.^28^ who found that in paediatric palliative research using parental perspectives, 75% of the respondents were mothers (p. 435). They write, ‘Gender can shape experiences of both parenthood and grief; balanced gender sampling and accurate analysis is essential for research on ‘parental perspectives’. Goodenough et al.^29^ showed associations between POD, gender of the parent and various psychological parameters, findings that cast doubt on the assumption that mothers and fathers have identical views on preferred POD.

### Response rate

Data on eligibility and enrolment are provided by five of the nine studies.^16,17,19–21^ The percentage of those eligible who actually participated ranges from 26% to 58%, mean = 43%. Four studies (mentioned earlier with the exception of Hechler et al.^16^) report the number whom they believe were successfully contacted. The range for those numbers is 35%–67%, mean = 55%. On average, 45% were not motivated to participate or unwilling to do so. Hechler et al.^16^ suggest that low rates may be connected with a cultural aversion to speak about death. In any case, the rates are low, and there is a real possibility that non-participants had a consistently different experience than those who came forward and participated in the study.

### Methodological issues within and across studies

The methods used to assess preferences in the studies reviewed vary widely. Those that were used were both too static and too rigid to allow for meaningful assessment of preference let alone to support policy development and allocation of resources based on preference in POD.

#### Place of care versus POD

There is some indication in these studies that what is the preferred place of care (POC) near the end of life may not be the preferred POD. Montel et al.^21^ mention that adolescents may prefer home for care and cite a case in which the child wanted to return to hospital 2 days before his death. Siden et al.^22^ mention that some families moved back and forth between settings. This could reflect not only a search for the best POD but also a family moving from a preferred POC to a preferred POD. Our clinical experience shows that families do make this distinction. For example, some parents want their child cared for at home but the death to be elsewhere – in a hospital or hospice. The literature reviewed does not truly grasp this; it has not uncovered what may be a common strategy that parents pursue, one that allows parents to reap the benefits of the best of different environments. This could mean that even at what is often called the end of life, when it is recognised that cure is no longer possible, there may be no single best place as parents try to manage the demands of care, the needs of the dying child and the siblings.

#### Undecided, change of preference and those who did not plan

It would seem fair to say that there is a presumption in many of these studies that the question of preference in POD, once confronted, is relatively straightforward. Significantly, however, several studies do not take this for granted and record a category of choice as ‘undecided’. The fraction expressing this undecided choice is not insignificant. Lyon et al.^18^ found that 20% expressed no preference, and Vickers et al.^23^ found at entry that 18% ‘had yet to express a preference’ (p. 4473). This fraction declined to 13% at a month before death.

A related finding is that in some studies, parents say they did not plan or choose their child’s POD. Montel et al.^21^ write, ‘Nineteen out of 21 (90%) families declared that they did not really choose their child’s POD. Death occurred suddenly before the parents had time to really make a decision’ (p. 30). Dussel et al.^20^ report that 52 of 140 (37%) families did not plan a location of death. Heath et al.^17^ report that 63% of parents felt that they had time in advance to plan their child’s POD. The average duration in the disease was 2.7 years, comparable to other studies reviewed.

If a goal of good healthcare provision is to support choice, that is, to provide and organise services in a way that allows participants to realise their choices, what do we make of those who are not making choices at all? Hannan and Gibson^30^ in a study of how parents determine the POC for children at the end of life observe, ‘Throughout this study it can be seen that families rarely seemed to make a conscious decision about where they wanted be, rather that the POC seemed obvious and natural’ (p. 287). This could suggest that changing POC and the absence of conscious decisions are natural and unproblematic.

These findings illustrate the design limitations of the current literature. The matrix of choices used to record preferences must accurately reflect the reality of the complexity of preference. Indeed, the basic model of how parents ‘make these decisions’ needs to be based upon empirical investigation and not constrained by a priori models, which embody untested ideas of a rational, responsible behaviour.

‘Evidence’ gathered through the application of inappropriate models is really not evidence at all. We need to understand the reasons for families’ uncertainty or indecision. Are they uncertain about how the disease will progress and what sort of care will be required? Are they evaluating and weighing the resources available? There are a number of reasons that we might put forward, including family coping strategies, family dynamics, past history of family illness, clinging to hopes of recovery and fears of the events surrounding death looming so large that considerations of preference and choice are obtrusive and unacceptable. To understand their indecision is to understand their needs.

#### The methods by which preferences are assessed

The methods used to determine preferences in the articles reviewed vary widely. In Grinyer and Thomas’^24^ study, there is no single method. Sometimes a conversation is interpreted as ‘showing’ a young adult’s wish. Sometimes a parent writes that it was their child’s wish to die in a particular location. Sometimes quotations attributed to the young person are provided in which the young person expresses a wish not to die in a particular location. It is not known whether these quoted remarks are taken from diaries or simply constructed from memories. Hechler et al.^16^ report that parents were asked which locale, in hindsight, was the most appropriate POD for their child. We do not know whether this was an open-ended question or whether parents chose between two or more options. With Lyon et al.,^18^ we do not know how the preferences were elicited. One assumes that the 31 items of the instrument were direct questions. Montel et al.’s^21^ statements are based on information elicited in semi-structured interviews. Vickers et al.^23^ tracked ‘planned POD’ on a monthly basis, presumably as part of medical record keeping. The locations given are ones ‘reported’. Clinical experience shows that this term can be interpreted broadly and could be, for example, the impression of the parents’ preference in the eyes of the staff member responsible for the records. Of all the studies, the study by Vickers et al.^23^ tracked in most detail, separating local hospital from United Kingdom Children’s Cancer Study Group (UKCCSG) tertiary centres and providing ‘other’ and ‘undecided’ as options.

Two of the studies infer preference from actual POD – the ‘reduced frequency of children dying in the hospital’ evidences a choice.^19,22^ In Siden et al.,^22^ it is important to note that they stress that families had equal access to all the three places of care. The data in Dussel et al.,^20^ which we used to infer preferences, consisted of a Likert scale question ‘How comfortable were you with the location of your child’s death?’ and a yes/no question about whether they would have preferred a different POD.

### Societal and cultural factors

Three articles mention cultural factors. Montel et al.^21^ state that ‘In France, as in most western countries, death is a subject of taboo’ (pp. 32, 35). Nineteen of the 21 families in the study did not discuss impending death with the CYP. The result was that ‘few families were actually offered this choice [of POD] (due to the difficulty of talking about death with the family)’. ‘It is a widely held belief in France that the hospital is the site of the best medical care’. This may explain why, in retrospect, all 19 families chose the hospital as POD.

Hechler et al.^16^ also mention cultural factors as having consequences for their study but by way of explaining the low response rate by parents. Vickers et al.^23^ write,

The proportion of children able to die at home in the UK is significantly greater than those indicated in studies in other countries (range, 49% to 60%). Many factors can affect parental decision making, including cultural differences. (p. 4475)

A societal/cultural issue is raised about the way in which a particular place is regarded or viewed. Montel et al.^21^ state that hospital is regarded in a certain way in France, and this has consequences for families’ behaviour. Surely, if one place has cultural import, then others do as well. It has been suggested that in Anglophone countries, the ‘default’ preference for POD is home.^31^ Preference for POD would not then be simply a function of the nature of disease and the type of care available. A variable that neither reflects nor is altered by the clinical environment is injected into the process.

If preference for POD is in part determined by culture, this underscores that what we are dealing with is precisely a value and not a judgement about the best place to die. As a cultural preference, it will also not be universal, even, say across Europe.

### Retrospection and recall

Six of the studies involved retrospective interviews. Heath et al.^17^ report mean time of death to interview as 4.5 years and Dussel et al.^20^ a median of 3.3; for Montel et al.^21^ and Hechler et al.,^16^ interviews did not commence for at least 2 years post death, and a mean of 3 years is plausible for them. Heath et al.^17^ raise the question of whether their results might be affected by recall bias and whether parents’ recollections were accurate some years after the events in question and ask whether parents’ ‘interpretations may have been affected by their own subsequent bereavement experiences’ (p. 74). Recall bias does not seem applicable here since it is generally used to mean a systematic difference in acuity of memory between cases and controls.^32^ Heath et al.’s^17^ other remark, however, is especially important because it acknowledges that many of these studies depend upon not just recall of isolated facts but also the interpretation of events. As bereaved, parents are hardly disinterested witnesses to the events in question. These are serious considerations that need to be addressed and, we believe, should temper any reliance on the reliability of the studies reviewed.

### The views of CYP themselves

Only one of the studies attempted to capture the views of YP themselves, whereas other studies attempted to capture their views from their parents, retrospectively: Grinyer and Thomas’^24^ narrative correspondence study. Clearly the preferences and more importantly the broader views of CYP about where they are cared for and where they want to die have not been studied directly. The obstacles to such studies are easy to understand. It would be difficult to secure ethics approval for the needed prospective studies with ill children, and additionally, recruitment may be inhibited both by HCP and parents wishing to shield their children from such conversations particularly in a research setting.

## Conclusion

What can be learned from a review of these nine articles? Clearly no synthesis of the findings to produce a more robust estimate of families’ preferences for POD for CYP is possible. Still, there are a number of important things to be gleaned.

We have noted earlier the support by government and by the voluntary sector for facilitating more home deaths. All the articles that we have included in this review support the position that whatever the majority of parents or families prefer, there is no one choice that is better or best.

One important question is whether attainment of preferred POD or death at home might serve as an outcome measure for palliative care. None of the studies reviewed, and a number of them make the point explicitly, provide any evidence to show that the quality of the experience of a home death, or hospital or hospice for that matter, is a better one. Indeed, Dussel et al.^20^ take the position that ‘the actual place of death may be less important than has been argued’ (pp. 34, 40). Their conclusion is that ‘the opportunity to plan location of death (LOD) may be a better proxy for high-quality end of life care than the actual LOD, one that is more inclusive and better aligned with palliative care principles’. Lowton^27^ writes that ‘focusing on *place* of death *per se* appeared to be of far less importance to parents than focusing on factors that influenced positively experiences of care at end-of-life’ (p. 1061). In a study of adults with cancer, Waghorn et al.^33^ found that dying at home was seventh (or sixth, depending) on a list of factors important for a good death. They conclude that POD may not be a good marker of quality at end of life.

Is preference for POD an important issue for future paediatric palliative care research? The experience of a death at home is a tremendously complex and varied experience. The articles reviewed, and the literature more broadly, show clearly that CYP can die at home, in hospital or in a hospice with the account given by their families describing their choice as one which met their needs and one which they did not regret. The important task ahead is, of course, to continue the search for factors more basic and general than POD, which allow us to reliably gauge the quality of families’ experience and of the care that has been rendered.
